# The TLR4-MyD88-NF-κB pathway is involved in sIgA-mediated IgA nephropathy

**DOI:** 10.1007/s40620-020-00722-3

**Published:** 2020-05-09

**Authors:** Junjun Zhang, Yiming Mi, Ruwen Zhou, Zhangsuo Liu, Bo Huang, Ruxue Guo, Panfei Wang, Yanru Lu, Yali Zhou, Songxia Quan

**Affiliations:** 1 Department of Nephrology, The First Affiliated Hospital of Zhengzhou University, Zhengzhou, 450052 People’s Republic of China; 2 Research Institute of Nephrology, Zhengzhou University, Zhengzhou, 450052 People’s Republic of China; 3 Key Laboratory of Precision Diagnosis and Treatment for Chronic Kidney Disease in Henan Province, Zhengzhou, 450052 People’s Republic of China; 4 Core Unit of National Clinical Medical Research Center of Kidney Disease, Zhengzhou, 450052 People’s Republic of China; 5 Department of Geriatric Medicine, The First Affiliated Hospital of Zhengzhou University, Zhengzhou, 450052 People’s Republic of China; 6 Department of Renal Pathology, The First Affiliated Hospital of Zhengzhou University, Zhengzhou, 450052 People’s Republic of China

**Keywords:** IgA nephropathy, Mucosal immunity, TLR4

## Abstract

Previous studies have shown that secretory IgA (sIgA) was critically involved in IgA nephropathy (IgAN) immune responses. Toll-like receptors (TLRs), especially TLR4 which participates in mucosal immunity, may be involved in the pathogenesis of IgAN. The purpose of this study was to investigate whether sIgA and TLR4 interact to mediate kidney damage in IgAN patients. IgAN patients with positive sIgA deposition in renal tissues were screened by immunofluorescence assay. Patient salivary sIgA (P-sIgA) was collected and purified by jacalin affinity chromatography. Salivary sIgA from healthy volunteers was used as a control (N-sIgA). Expression of TLR4, MyD88, NF-κB, TNF-α, IL-6, and MCP-1 were detected in the mesangial area of IgAN patients by immunohistochemistry, the expression levels in patients with positive sIgA deposition were higher than that with negative sIgA deposition. Human renal mesangial cells (HRMCs) were cultured in vitro, flow cytometry showed that P-sIgA bound HRMCs significantly better than N-sIgA. HRMCs were cultured in the presence of sIgA (400 μg/mL) for 24 h, compared with cells cultured with N-sIgA, HRMCs cultured in vitro with P-sIgA showed enhanced expression of TLR4, increased secretion of TNF-α, IL-6, and MCP-1, and increased expression of MyD88/NF-κB. TLR4 shRNA silencing and NF-κB inhibition both reduced the ability of HRMCs to synthesize TNF-α, IL-6, and MCP-1. Our results indicate that sIgA may induce high expression of TLR4 in HRMCs and further activate downstream signalling pathways, prompting HRMCs to secrete multiple cytokines and thereby mediating kidney damage in IgAN patients.

## Introduction

IgA nephropathy (IgAN) is the most common primary glomerulonephritis in China and worldwide. IgAN is detected in about 50% of kidney biopsies in Asia and up to 45% of biopsies in China [1, 2]. Approximately 20–40% of IgAN patients progress to end-stage renal disease within 20 years [3]. IgAN mainly affects young people, which imposes a serious burden on families and society. Therefore, research on IgAN is important and necessary.

Diagnosis of IgAN relies on immunopathology. The disease is characterized by deposition of IgA-based immune complexes in the glomerular mesangial areas which can be observed using immunofluorescence assays. At present, it is believed that loss of IgA glycosylation, genetic factors and mucosal immunity are all involved in the pathogenesis of IgAN [4, 5]. The detailed pathogenesis of IgAN is still not fully understood. A series of previous studies suggested that mucosal immune abnormalities, especially of sIgA, are involved in the development of IgAN. Our previous work showed that about one third of IgAN patients showed deposition of sIgA in renal tissue, and that sIgA in IgAN patients is pathogenic, contributing to the proliferation and activation of human renal mesangial cells (HRMCs) [6–8]. Moreover, multiple miRNAs are involved in regulation of sIgA-induced HRMC cytokine secretion and subsequent renal injury [9]. These studies suggest a key role for sIgA in IgAN.

In recent years, many studies have suggested that Toll-like receptors (TLRs), especially TLR4 which is important for mucosal immunity, may be involved in the pathogenesis of IgAN. TLRs play a crucial role in the innate immune response to invading pathogens by recognizing conserved pathogen-associated molecular patterns. TLRs play a crucial role in linking the innate and adaptive immune systems, and are distributed on the surfaces of different immune cells [10]. TLR4 is highly expressed in renal tissue of patients with IgAN [11]. Coppo et al. found that expression of TLR4 and abundance of mRNA transcripts encoding TLR4 in peripheral blood mononuclear cells were significantly higher in IgAN patients compared with healthy controls. TLR4 expression levels were closely associated with haematuria and 24-h urine protein quantitation [12]. Recent studies have shown that TLR4 leads to mesangial cell damage by inducing production of pro-inflammatory factors, leading to the development of IgAN [13]. These studies suggest that TLR4 plays an important role in the pathogenesis of IgAN.

Based on our previous findings and other published work, we hypothesized that sIgA may lead to cytokine production by inducing high expression of TLR4 in HRMCs, thus promoting IgAN kidney damage. This study was designed to test this hypothesis.

## Materials and methods

### Patients

We enrolled 87 renal biopsy-confirmed primary IgAN patients from our department. IgAN diagnoses were confirmed by observation of granular IgA deposition with absence of IgM deposition in the glomerular mesangium via an immunofluorescence assay. Patients with Henoch-Schönlein purpura, systemic lupus erythematosus, diabetes mellitus, active hepatitis, liver cirrhosis, severe metabolic syndrome and secondary IgAN-related diseases were excluded.

### Ethics statement

The study was reviewed and approved by the Ethics Committee of the First Affiliated Hospital of Zhengzhou University, and was carried out according to the principles laid out in the World Medical Association’s Declaration of Helsinki.

### Detection of sIgA deposition by immunofluorescence assay

Deposition of sIgA in renal tissue was detected by immunofluorescence assay as previously described [6]. Briefly, the primary antibody was a mouse monoclonal antibody against human secretory component (SC) (1:100 dilution; Genetex, San Antonio, CA). Kidney tissue sections were incubated overnight at 4 °C with the primary antibody. The secondary antibody was Alexa Fluor 594-conjugated donkey anti-mouse IgG (1:100, Invitrogen, Paisley, UK). Sections were incubated for 60 min at 37 °C with the secondary antibody. Fluorescein isothiocyanate (FITC)-labeled rabbit anti-human IgA (polyclonal, 1:30 dilution; Dako, Glostrup, Denmark) was then added at 37 °C for 60 min. All sections were observed under confocal microscopy (LSM 710; Zeiss, Oberkochen, Germany). The primary antibody was replaced with phosphate-buffered saline (PBS) as a negative control.

### Detection of TLR4, MyD88, NF-κB, tumor necrosis factor (TNF)-α, interleukin (IL)-6 and macrophage chemoattractant protein (MCP)-1 expression in kidneys by immunohistochemistry

Paraffin-embedded renal tissue sections were used for immunohistochemistry using anti-TLR4 (Santa Cruz Biotechnology, Santa Cruz, CA, USA), -MyD88, and -NF-κB p65 (Cell Signaling Technology, CA, USA) primary antibodies. PBS was used as a negative control. Antibodies against TLR4 were diluted in 0.01 M PBS, pH 7.4. Renal staining for TLR4, MyD88 and NF-κB was evaluated using Image Pro Plus software version 6.0. Positive signals were quantified as mean optical density.

### Saliva collection and isolation of sIgA

Saliva was collected from IgAN patients with positive sIgA deposition as previously described [8]. After centrifugation at 4 °C, the supernatant was stored at − 80 °C. We collected saliva from age- and gender-matched healthy volunteers with no recent history of mucosal infection or kidney disease as controls.

Patient salivary sIgA (P-sIgA) and healthy control salivary sIgA (N-sIgA) were purified by jacalin affinity chromatography as previously described [8].

### Cell culture and treatments

HRMCs were cultured according to the manufacturer's recommendations (ScienCellTM, Carlsbad, CA, USA). HRMCs were starved in mesangial cell medium (MCM) without foetal bovine serum (FBS) for 24 h, then stimulated with 400 μg/mL P-sIgA or N-sIgA for 24 h. Controls were incubated with MCM without FBS.

The supernatants were collected and stored at − 80 °C after centrifugation until subsequent experiments.

### Flow cytometry

HRMCs were grown to log phase and harvested using 0.05% trypsin/0.02% ethylenediaminetetraacetic acid for 2 min at 37 °C. Cell were counted using a haemocytometer and an average of 1 × 10^5^ cells/well were used. Staining was performed at 4 °C. The cells were incubated with sIgA (final concentration: 400 μg/mL) for 30 min. Cells were washed with Dulbecco’s PBS and then further incubated with 10 μL of FITC-conjugated goat anti-human IgA antibody. The stained cells were analysed using a BD FACS Calibur (BD, USA). A minimum of 1000 fixed cells for each sample were analysed.

### Transfection and inhibition experiments

HRMCs were seeded in six-well plates 24 h prior to viral infection. The cells were incubated overnight in complete MCM containing FBS and antibiotics, and were approximately 50% confluent the next day. Complete medium containing Polybrene (Santa Cruz, USA), a polycation that neutralizes charge interactions to increase binding between the pseudoviral capsid and the cell membrane, was prepared. The TLR4 shRNA lentiviral particles (Santa Cruz, USA) were added to cells and incubated overnight. Control shRNA lentiviral particles (Santa Cruz, USA) were used as a control. On the third day, the culture medium was removed and replaced with complete MCM without Polybrene. For stable transduction, transduced cells were cultured in puromycin dihydrochloride (Santa Cruz, USA) until the next experiment. Western blotting and RT-PCR were used to verify the efficiency of infection. HRMCs were pretreated for 90 min with BAY 11-7082 (Sigma, USA) to block NF-κB signaling. The HRMCs were then stimulated with purified sIgA.

### ELISA

For detection of TNF-a, IL-6 and MCP-1 in cell culture supernatants, standard sandwich ELISA assays were performed using human TNF-a, IL-6 and MCP-1 ELISA kits (R&D Systems, Minneapolis, MN, USA).

### Western blotting

HRMCs were homogenized in lysis buffer and boiled at 100 °C for 5 min. Each total protein sample (20 μg) was separated by electrophoresis on 10% SDS-PAGE gels and transferred onto a polyvinylidene difluoride (PVDF) membrane. After blocking with 5% skim milk, the membranes were incubated with mouse anti-TLR4 antibody (1:500, Santa Cruz Biotechnology, USA), rabbit anti-MyDBB antibody (1:1000, Cell Signaling Technology, USA), rabbit anti-NF-κB P65 antibody (1:1000, Cell Signaling Technology, USA), or mouse anti-β-actin antibody (1:1000 Santa Cruz Biotechnology, USA) overnight at 4 °C. After washing three times every 5–10 min with Tris-buffered containing 0.1% (v/v) Tween-20 (TBST), the PVDF membranes were incubated with horseradish peroxidase-conjugated goat anti-rabbit IgG or goat anti-mouse IgG antibodies (1:5000, Proteintech, China) for 1 h at room temperature. After another three washes with TBST, blots were developed and imaged using an enhanced chemiluminescence detection system (FluorChem E, proteinsimple, USA).

### Quantitative PCR (qPCR)

Total cellular RNA was extracted using TRIZOL^®^ Reagent (Invitrogen, Carlsbad, CA, USA) according to the manufacturer’s instructions and reverse transcribed into cDNA using SuperScript™ II Reverse Transcriptase (Invitrogen). The cDNA was stored at − 20 °C until amplification. Abundance of mRNA transcripts was quantified by qPCR with the Prism 7500 sequence detecting system (ABI, USA) using the SYBR PrimeScript RT-PCR Kit in 20-μL reaction volumes (Takara Bio, Japan) according to the manufacturer’s protocols. Relative mRNA expression was calculated with the comparative ΔΔCT method using the formula: relative expression = 2^−ΔΔCT^.

The primer sequences used are shown in Table 1.

**Table 1:** Sequences of oligonucleotide primers used in this study

Gene name	Forward primer (5′–3′)	Reverse primer (5′–3′)
TLR4	TTTGGACAGTTTCCCACATTGA	AAGCATTCCCACCTTTGTTGG
NF-κB	ATGTGGAGATCATTGAGCAGC	CCTGGTCCTGTGTAGCCATT
MyD88	GGCTGCTCTCAACATGCGA	CTGTGTCCGCACGTTCAAGA
MCP-1	CAGCCAGATGCAATCAATGCC	TGGAATCCTGAACCCACTTCT
TNF-a	CCTCTCTCTAATCAGCCCTCTG	GAGGACCTGGGAGTAGATGAG
IL-6	ACTCACCTCTTCAGAACGAATTG	CCATCTTTGGAAGGTTCAGGTTG
ACTB	CCTGGCACCCAGCACAAT	GCTGATCCACATCTGCTGGAA

### Statistical analyses

Statistical analyses were performed using SPSS software (version 20.0; SPSS, Chicago, USA). Normally distributed data were summarized as means ± standard deviations (SDs). Independent-sample t-tests were used to analyse differences between two groups. A *p* value < 0.05 was considered significant.

## Results

### Deposition of SIgA in renal tissue of patients with IgAN

To examine the deposition of SIgA in the renal tissue of IgAN patients, we used immunofluorescence to detect the deposition of IgA and SC. IgA deposition was detected in all 87 patients, 27 of whom had SC deposition at the same time (result reference to [6]). The results indicate that approximately one-third of IgAN patients have deposition of SIgA.

### Binding of sIgA to HRMCs

**Fig. 1:**
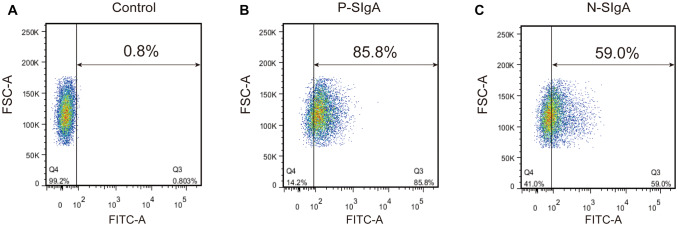
Flow cytometry to assess sIgA binding to HRMCs. **a** PBS as a negative control. **b** Binding of N-sIgA to HRMCs was increased when compared with control subjects. **c** Binding of P-sIgA to HRMCs was increased when compared with N-sIgA

To investigate the mechanism of sIgA deposition in the mesangial region, we examined binding of sIgA to HRMCs by flow cytometry. After incubation of HRMCs with FITC-conjugated sIgA, binding was assessed. P-sIgA stained approximately 85% of HRMCs (Fig. 1b), significantly higher than N-sIgA (Fig. 1c).

### TLR4, TNF-α, IL-6 and MCP-1 immunohistochemistry

**Fig. 2:**
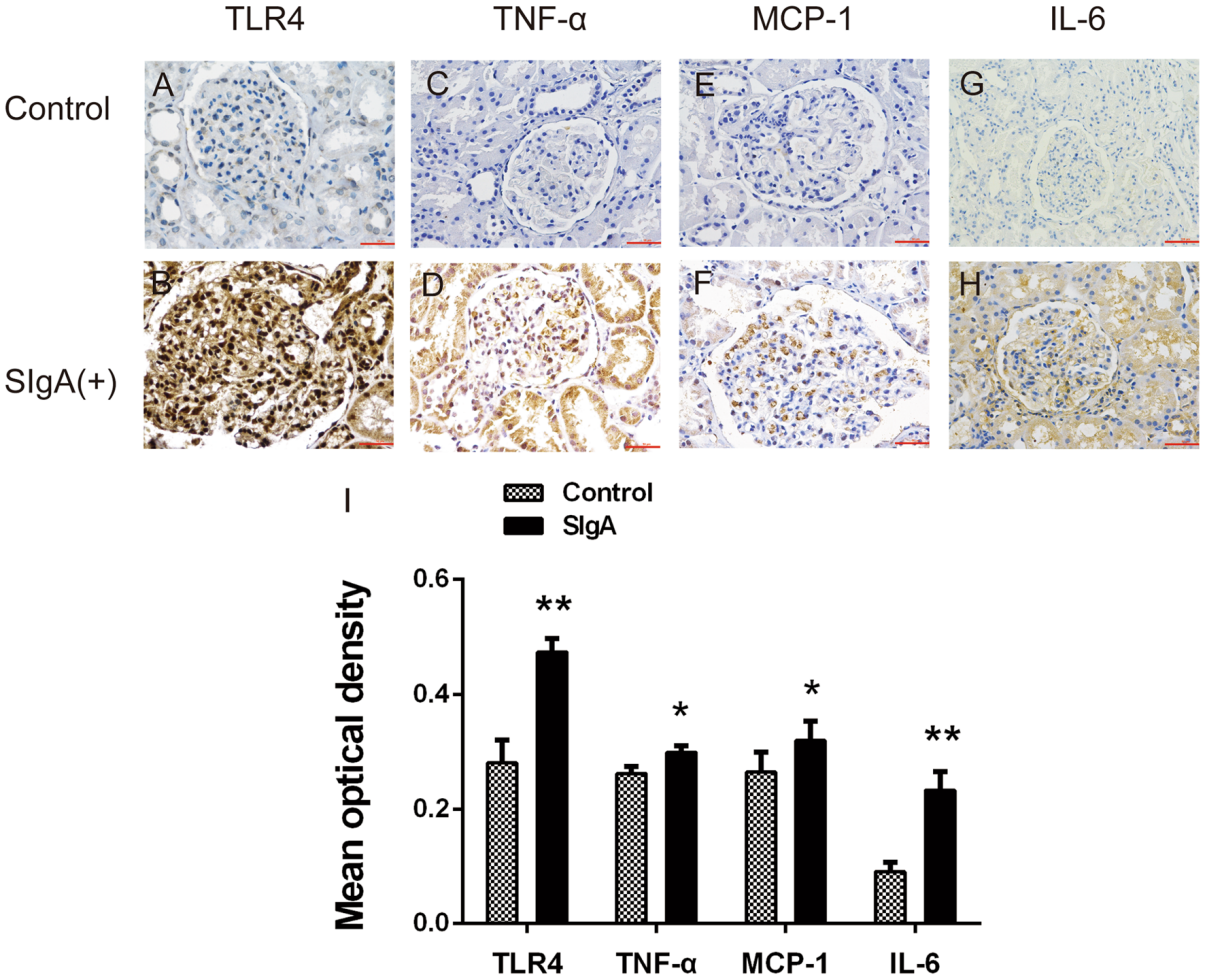
Immunohistochemical staining of TLR4, TNF-α, IL-6 and MCP-1 in glomerular mesangial areas of kidney specimens. **a**, **c**, **e**, **g** Immunohistochemical staining of TLR4, TNF-α, IL-6 and MCP-1 in glomerular mesangial areas of normal controls; **b**, **d**, **f**, **h** Immunohistochemical staining of TLR4, TNF-α, IL-6 and MCP-1 in glomerular mesangial areas of IgAN patients with positive sIgA deposition. **i** Analysis of the mean optical density of TLR4, TNF-α, IL-6 and MCP-1(*p < 0.05, **p < 0.01)

In the kidneys of IgAN patients with positive sIgA deposition, immunohistochemistry detected increased expression of TLR4, TNF-α, IL-6 and MCP-1 in glomerular mesangial areas. Expression was low in the glomerular mesangial areas of normal controls. The mean optical densities of TLR4, TNF-α, IL-6 and MCP-1 in the glomerular mesangial areas of IgAN patients with positive sIgA were significantly higher than those of normal controls (Fig. 2).

### sIgA induces high expression of TLR4 and increased synthesis of TNF-α, IL-6 and MCP-1 in HRMCs

**Fig. 3:**
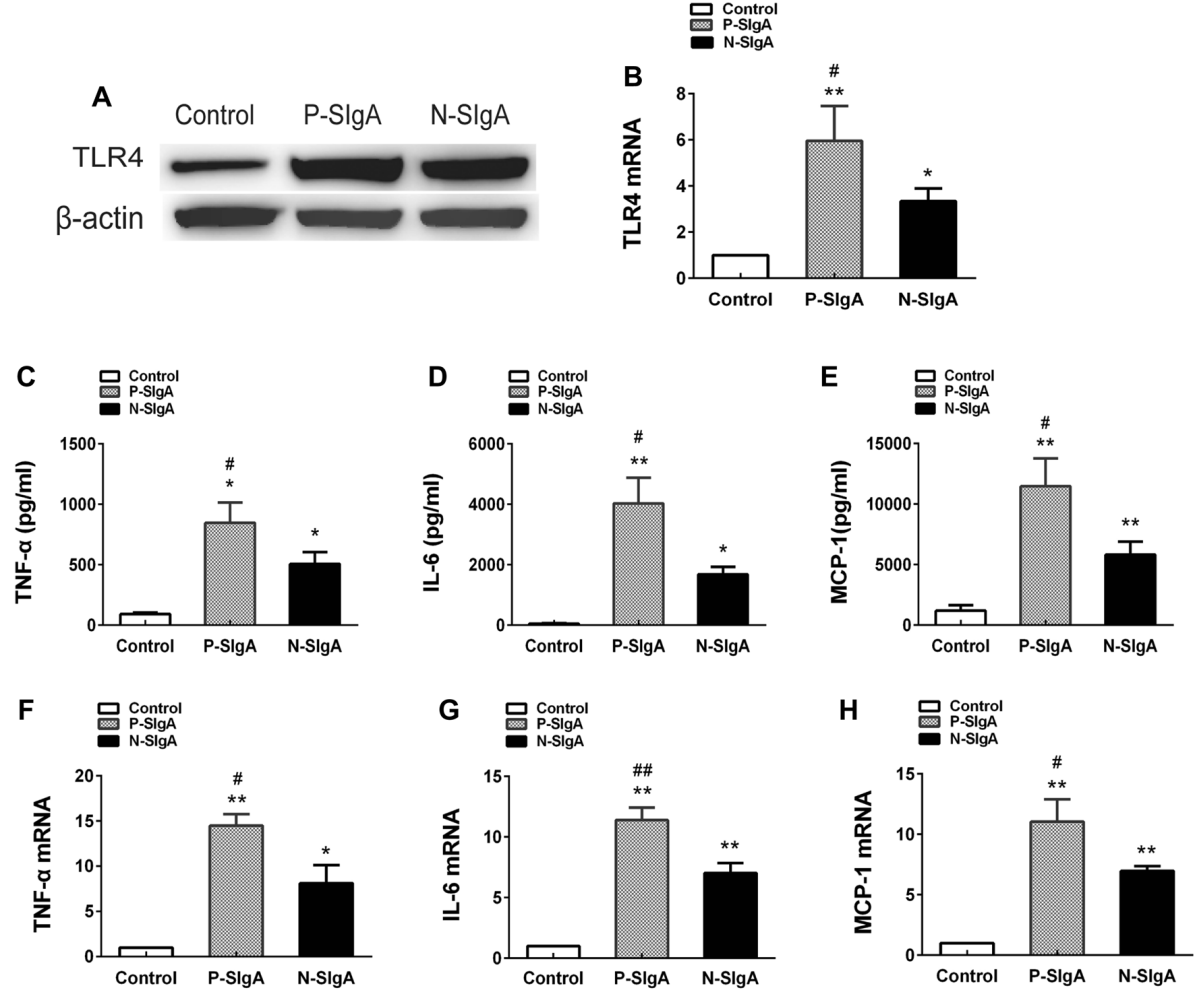
Protein and mRNA abundance after stimulation of HRMCs with P-sIgA, N-sIgA or PBS (negative control). **a** Western blotting analysis of TLR4 expression, with β-actin as a control. **b** Abundance of mRNA transcripts encoding TLR4 as determined by qPCR, with β-actin as a control. **c**, **e** Levels of TNF-α, IL-6 and MCP-1 expression in cell supernatants as determined by ELISA. **f**–**h** Abundance of mRNA transcripts encoding TNF-α, IL-6 and MCP-1 as determined by qPCR, with β-actin as a control. All experiments were performed in triplicate. Results are shown as means ± SDs (*p < 0.05, ^#^*p* < 0.05, **p < 0.01, ^##^*p* < 0.01)

To investigate the effect of the interaction between sIgA and TLR4 on HRMCs, we stimulated HRMCs with sIgA (400 μg/mL) for 24 h. Persistent stimulation of HRMCs resulted in increased expression of TLR4, TNF-α, IL-6 and MCP-1 at the protein and mRNA levels. Expression increases were more dramatic in P-sIgA-treated cells than N-sIgA-treated cells (*p* < 0.05), and both were higher than the negative control group (*p* < 0.05) (Fig. 3).

### TLR4 shRNA lentiviral particles (shTLR4) attenuated sIgA-induced production of TNF-α, IL-6 and MCP-1

**Fig. 4:**
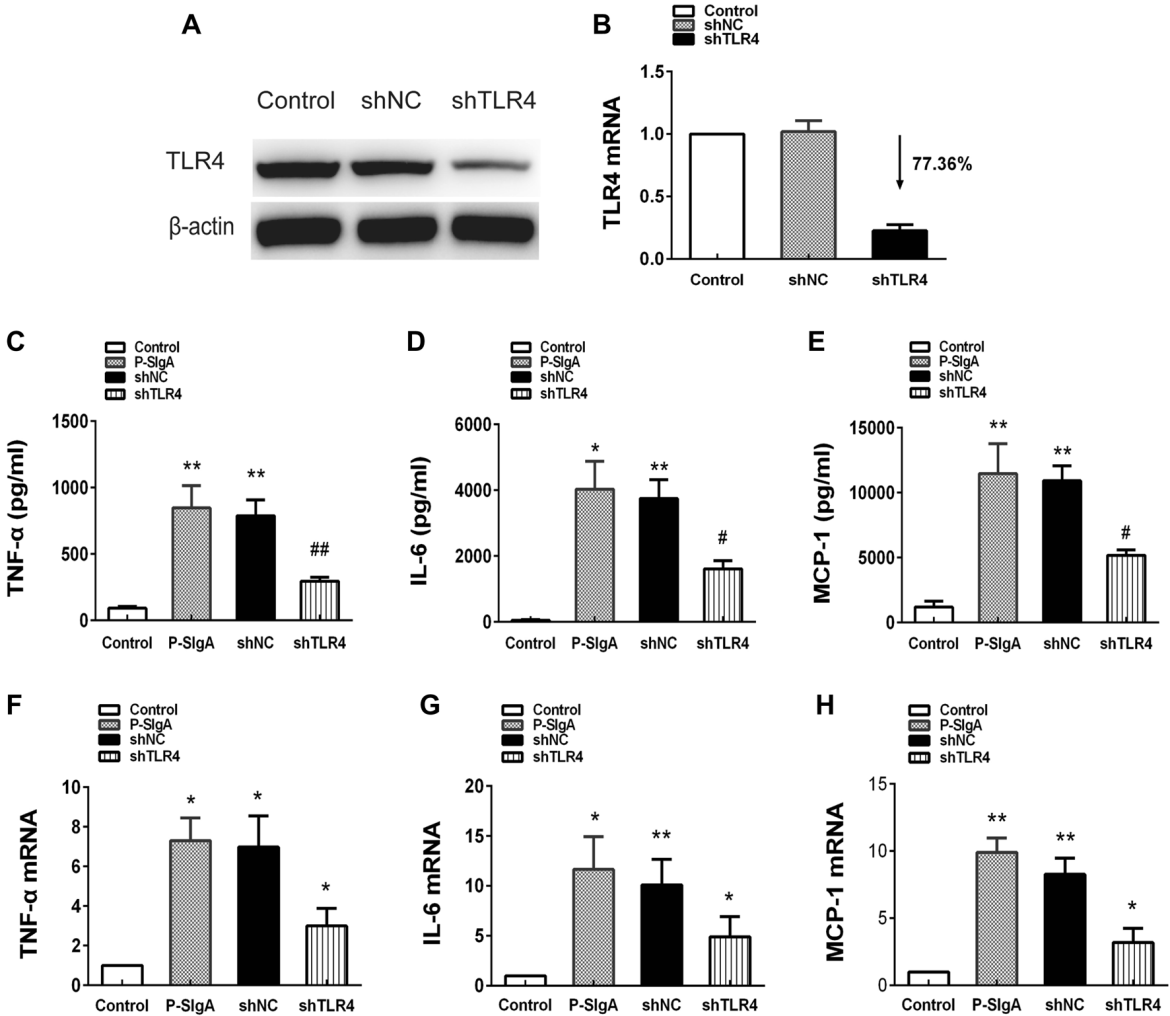
**a**, **b** Transfection of HRMCs with negative control shRNA lentiviral particles (shNC) or TLR4 shRNA lentiviral particles (shTLR4) followed by qPCR analysis of TLR4 mRNA abundance. The shTLR4 silencing efficiency was 77%. **c**–**e** HRMCs were transfected with shNC or shTLR4 and then stimulated with sIgA (400 μg/mL) for 24 h. ELISA was used to assess expression of TNF-α, IL-6, MCP-1. **f**–**h** Quantitation of TNF-α, IL-6 and MCP-1 mRNA abundance, with β-actin as a control. All experiments were performed in triplicate. Values were expressed as means ± SDs (*p < 0.05, ^#^*p* < 0.05, **p < 0.01, ^##^*p* < 0.01)

To further explore the role of TLR4 in regulation of sIgA-induced TNF-α, IL-6 and MCP-1 production, we used shTLR4 to silence TLR4 expression in HRMCs. We observed significant differences in TLR4 expression by HRMCs transfected with shTLR4 and a negative control shRNA (shNC), and the silencing efficiency was 77% (*p* < 0.05, Fig. 4). After transfection, HRMCs were stimulated with sIgA (400 μg/mL) for 24 h. TNF-α, IL-6 and MCP-1 protein synthesis and mRNA expression were decreased, indicating that that TLR4 is involved in regulating sIgA-induced synthesis of TNF-α, IL-6 and MCP-1 (Fig. 4).

### MyD88 and NF-κB immunohistochemistry

**Fig. 5:**
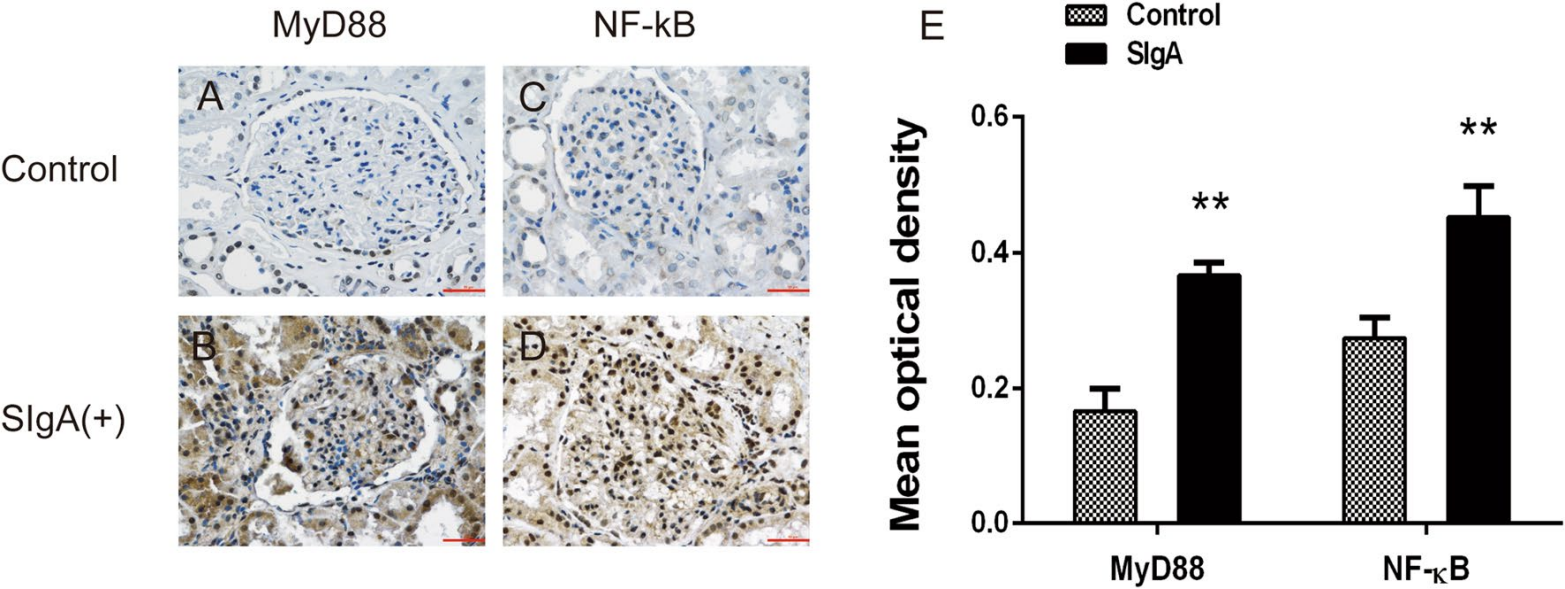
Immunohistochemical staining of MyD88 and NF-κB in glomerular mesangial areas. **a**, **c** Immunohistochemical staining of MyD88 and NF-κB in glomerular mesangial areas of healthy controls. **b**, **d** Immunohistochemical staining of MyD88 and NF-κB in glomerular mesangial areas of IgAN patients with positive sIgA deposition. **e** Analysis of the mean optical density of MyD88 and NF-κB (*p < 0.05, **p < 0.01)

In the kidneys of IgAN patients with positive sIgA deposition, immunohistochemistry showed increased expression of MyD88 and NF-κB in glomerular mesangial areas but low expression in the glomerular mesangial areas of normal controls. The mean optical densities for MyD88 and NF-κB in the glomerular mesangial areas of IgAN patients with positive sIgA deposition were significantly higher than those of normal controls (Fig. 5).

### sIgA induces high expression of MyD88 and NF-κB in HRMCs

**Fig. 6:**
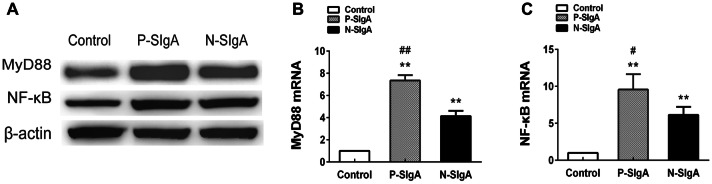
Protein and mRNA abundance after stimulation of HRMCs with P-sIgA, N-sIgA or PBS (negative control). **a** Western blotting analysis of MyD88 and NF-κB expression, with β-actin as a control. **b**, **c** Abundance of mRNA transcripts encoding MyD88 and NF-κB as determined by qPCR, with β-actin as a control. All experiments were performed in triplicate. Results are shown as means ± SDs (*p < 0.05, ^#^*p* < 0.05, **p < 0.01, ^##^*p* < 0.01)

We stimulated HRMCs with sIgA (400 μg/mL) for 24 h. Persistent stimulation of HRMCs resulted in increased expression of MyD88 and NF-κB at the protein and mRNA levels. Expression was increased in P-sIgA-treated cells more significantly than in N-sIgA-treated cells (*p* < 0.05). Both groups of treated cells showed elevated expression compared with the negative control group (*p* < 0.05) (Fig. 6).

### Inhibition of sIgA-induced TNF-α, IL-6 and MCP-1 production by the NF-κB inhibitor BAY 11-7082

**Fig. 7:**
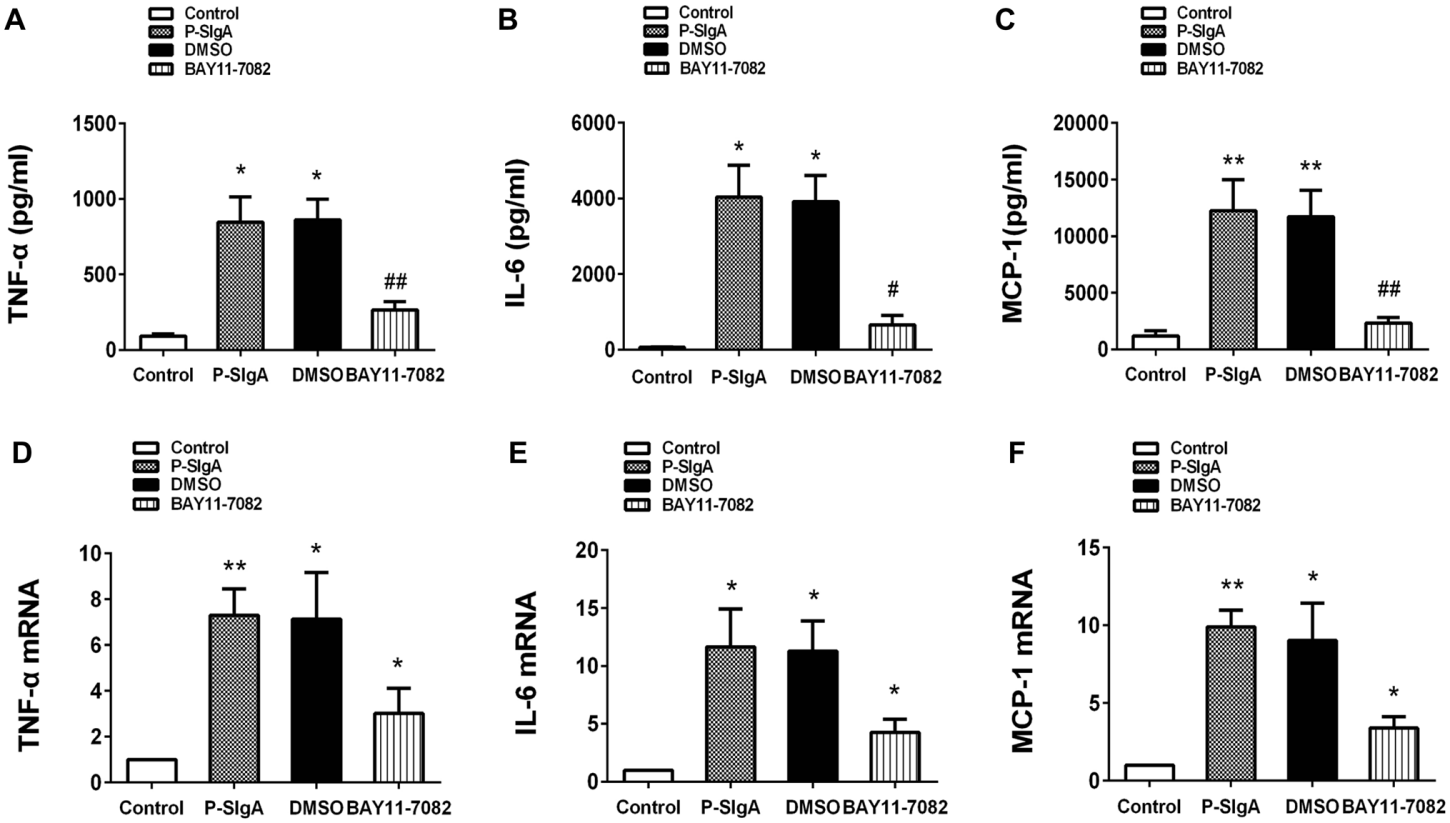
HRMCs were pre-treated with the NF-κB inhibitor BAY 11-7082 (5 μM) for 90 min and then stimulated with sIgA (400 μg/mL) for 24 h. Dimethyl sulfoxide was used as a negative control. **a**–**c** The levels of TNF-α, IL-6 and MCP-1were analyzed by ELISA. **d**–**f** Quantitation of mRNA transcripts encoding TNF-α, IL-6 and MCP-1, with β-actin as a control. All experiments were performed in triplicate. Values were expressed as means ± SDs (*p < 0.05, ^#^*p* < 0.05, **p < 0.01, ^##^*p* < 0.01)

To understand the role of NF-κB in sIgA-induced TNF-α, IL-6 and MCP-1 production, a small-molecule NF-κB inhibitor (BAY 11-7082) was used. HRMCs were seeded in six-well plates at a density of 1 × 10^6^ cells/well. The cells were pre-treated with BAY 11-7082 for 2 h and then treated with sIgA for 24 h. BAY11-7082 significantly decreased the expression of TNF-α, IL-6 and MCP-1 induced by sIgA compared with the control group (*p* < 0.05). Thus, in HRMCs, sIgA-induced TNF-α, IL-6 and MCP-1 production occurs in a NF-κB-dependent fashion (Fig. 7).

## Discussion

IgAN was first described in 1968 by the French pathologists Berger and Hinglais [14, 15]. It is the most common type of human primary glomerulonephritis worldwide. The role of mucosal immunity and sIgA in the pathogenesis of IgAN has gradually gained attention. sIgA is the most important antibody isotype involved in mucosal immunity, and is mainly produced by mucosal lymphoid tissue B cells and secreted into the oral cavity, intestinal digestive juice, respiratory mucosa, colostrum, and to a lesser extent, the blood [16, 17]. Patients with IgAN often display gross haematuria and/or proteinuria following upper respiratory tract or intestinal tract infections [18, 19]. Genome-wide association studies (GWAS) showed that genes associated with IgAN risk are also associated with mucosal immunity [20–24]. Immunization with mucosal-associated antigens can induce IgAN in animal models [25]. The NEFIGAN study found that targeted release of budesonide to distal intestinal lymphoid tissue reduced the ratio of urinary protein to creatinine in IgAN patients [26, 27]. Our previous study showed that sIgA plays an important role in the pathogenesis of IgAN. The pathogenesis of IgAN has not yet been fully clarified, but Suzuki's “four-hit” doctrine has been recognized by many researchers [28]. An important step is the deposition of IgA in mesangial areas leading to cytokine production, interstitial fibrosis and a series of other changes. Of the IgA deposited in the mesangial area, about two thirds is polymeric IgA (pIgA) from serum and one third is sIgA. However, most previous studies focused on damage to HRMCs caused by pIgA [29–31], and the effect of sIgA on HRMCs has rarely been discussed. Our previous study found that sIgA in IgAN patients is pathogenic, stimulating HRMC proliferation and activating HRMCs to secrete a variety of pro-inflammatory, pro-proliferative, and pro-fibrotic factors. These factors can then participate in kidney damage. Therefore, sIgA may play an important role in IgAN pathogenesis.

In recent years, the role of TLR4 in the pathogenesis of IgAN has been widely studied. In kidney disease, TLR4 is highly expressed in the renal tissues of IgAN patients, and may be involved in damage to murine mesangial cells by promoting the production of pro-inflammatory factors [32]. Some researchers have also found that hydroxychloroquine effectively reduces proteinuria in patients with IgAN by reducing the expression of TLR4 [33, 34]. These studies suggest that TLR4 plays an important role in IgAN. In our previous studies, we found that sIgA caused HRMCs to proliferate and secrete cytokines [6–9]. Therefore, we speculated that sIgA may induce cytokine production via upregulation of TLR4 in HRMCs, which may further cause IgAN kidney damage. To confirm this hypothesis, we first tested the binding of sIgA to HRMCs by flow cytometry. About one third of IgAN patients showed sIgA deposition in renal tissue, and P-sIgA stained up to 85% of HRMCs. By contrast, N-sIgA stained HRMCs to a much lesser extent. Our results suggest that sIgA in IgAN may be deposited in mesangial areas by binding to HRMCs and further interacting with these cells. Oortwijn et al. also found that sIgA (purified from human colostrum and purchased from Sigma) can bind to HRMCs in vitro [35]. Although our sources of sIgA were different, the data all indicate that sIgA and HRMCs can interact in vitro.

However, previous studies could not determine whether the interaction between sIgA and TLR4 led to the production of cytokines. To further clarify this issue, we assessed the expression of TLR4 and various cytokines in kidney tissue. Immunohistochemical results showed that expression of TLR4, TNF-a, IL-6 and MCP-1 in renal tissues of patients with sIgA-positive IgAN were significantly enhanced. Next, we stimulated HRMCs with sIgA and assessed the expression of TLR4 and inflammatory factors. Following stimulation with sIgA, expression of TLR4 was increased in HRMCs, and secretion of TNF-a, IL-6 and MCP-1 was also increased. Previous studies reported that TLR4 is expressed by HRMCs, and high expression of TLR4 may be associated with inflammatory factor production and renal fibrosis [36–40]. For example, IL-6 and MCP-1 induce proliferation of mesangial cells and have a pro-inflammatory effect on IgAN glomerular injury [41–43]. IgA deposition in the mesangial area of IL-6 knockout mice was reduced [44]. MCP-1 induced mesangial cell proliferation and matrix deposition, and further aggravated renal damage [45–49]. TNF-α may be involved in IgAN crescent formation and is associated with the severity of renal interstitial fibrosis [30, 50–53], and GWAS indicated that TNF-α-related genes were associated with IgAN [54–56]. To further investigate the relationship between cytokine production and TLR4, we used TLR4 shRNA lentiviral transfection to silence TLR4 expression in HRMCs. We found that the expression of all three cytokines was significantly reduced in TLR4-silenced cells, indicating that cytokine production can be regulated by TLR4. However, we found that production of cytokines was not completely blocked, indicating that their production may also be regulated by other pathways. Thus, above studies suggest that sIgA, TLR4 and cytokines play an important role in the pathogenesis of IgAN. Our results indicate that sIgA can induce high expression of TLR4 in HRMCs, promoting cytokine production and leading to kidney damage.

Previous studies have shown that TLR4 plays a role in inflammatory and fibrotic processes in renal diseases, which are usually associated with activation of the MyD88-NF-κB pathway. The NF-κB pathway is closely linked with inflammatory responses during kidney disease and renal tissue damage [36–38, 46, 47, 53, 57, 58]. Choi et al. found that production of cytokines such as MCP-1 and IL-8 in glomerular diseases are associated with the activation of NF-κB [58]. Lai et al. showed that TNF-α production in IgAN patients is regulated by NF-κB [57], and recent studies have shown that activation of the NF-κB pathway is closely associated with renal fibrosis [59, 60]. We examined the expression of signalling proteins in renal tissues, and found that MyD88 and NF-κB were highly expressed in the renal tissues of sIgA-positive IgAN patients. To further investigate the mechanism through which sIgA stimulates HRMCs to induce high expression of TLR4 and cytokine release, we assessed signalling pathways and the effects of their inhibition. First, we examined the expression of MyD88 and NF-κB following sIgA stimulation of HRMCs. Expression of MyD88 and NF-κB at the protein and mRNA levels was significantly increased in P-sIgA-treated cells. Next, we used an NF-κB signalling inhibitor (BAY 11-7082) to block NF-κB transduction in HRMCs and found that expression of TNF-α, IL-6 and MCP-1 was significantly reduced. Therefore, our results demonstrate that the production of TNF-a, IL-6 and MCP-1 is regulated to some extent by the MyD88-NF-κB pathway.

In conclusion, our study found that sIgA may be deposited in mesangial areas by binding to HRMCs, inducing high expression of TLR4 in mesangial cells and further activating the MyD88-NF-κB signalling pathway. Finally, signalling leads to the production of TNF-a, IL-6 and MCP-1 and damage to the kidneys. This finding may provide new insights into the pathogenesis and treatment of IgAN.
